# Changes of Bone Turnover Markers in Long Bone Nonunions Treated with a Regenerative Approach

**DOI:** 10.1155/2017/3674045

**Published:** 2017-06-20

**Authors:** Donatella Granchi, Enrique Gómez-Barrena, Markus Rojewski, Philippe Rosset, Pierre Layrolle, Benedetta Spazzoli, Davide Maria Donati, Gabriela Ciapetti

**Affiliations:** ^1^Orthopedic Pathophysiology and Regenerative Medicine Unit, Rizzoli Orthopedic Institute, Bologna, Italy; ^2^Hospital La Paz, IdiPAZ, Universidad Autónoma de Madrid, Madrid, Spain; ^3^Institute for Clinical Transfusion Medicine and Immunogenetic Ulm (IKT Ulm), Ulm, Germany; ^4^Service of Orthopaedic Surgery and Traumatology, CHRU, Tours, France; ^5^Inserm U957, Laboratoire de Physiopathologie de la Résorption Osseuse et Thérapie des Tumeurs Osseuses Primitives (LPRO), University of Nantes, Nantes, France; ^6^III Orthopedic and Traumatology Clinic, Rizzoli Orthopedic Institute, Bologna, Italy; ^7^Department of Biomedical and Neuromotor Sciences, University of Bologna, Bologna, Italy

## Abstract

In this clinical trial, we investigated if biochemical bone turnover markers (BTM) changed according to the progression of bone healing induced by autologous expanded MSC combined with a biphasic calcium phosphate in patients with delayed union or nonunion of long bone fractures. Bone formation markers, bone resorption markers, and osteoclast regulatory proteins were measured by enzymatic immunoassay before surgery and after 6, 12, and 24 weeks. A satisfactory bone healing was obtained in 23 out of 24 patients. Nine subjects reached a good consolidation already at 12 weeks, and they were considered as the “early consolidation” group. We found that bone-specific alkaline phosphatase (BAP), C-terminal propeptide of type I procollagen (PICP), and beta crosslaps collagen (CTX) changed after the regenerative treatment, BAP and CTX correlated to the imaging results collected at 12 and 24 weeks, and BAP variation along the healing course differed in patients who had an “early consolidation.” A remarkable decrease in BAP and PICP was observed at all time points in a single patient who experienced a treatment failure, but the predictive value of BTM changes cannot be determined. Our findings suggest that BTM are promising tools for monitoring cell therapy efficacy in bone nonunions, but studies with larger patient numbers are required to confirm these preliminary results.

## 1. Introduction

Normal fracture healing in adults occurs through intramembranous or endochondral bone formation, depending on the primary stability of the fracture site: if stability is maintained through a rigid fixation and a perfect reduction, the healing may proceed through an intramembranous mechanism with limited cartilaginous callus formation [1]. Bone healing after fracture is a complex process finely tuned where several types of cells, factors, and molecules cooperate locally to restore tissue continuity and function. The “biology” of bone repair, that is, the progression of biochemical and molecular signals during healing of bone, has been described by several authors [2, 3]. The ability to assess cells and molecules involved in bone turnover during fracture healing would aid in monitoring the reparative process and in predicting the treatment outcome. Despite the ability of bone for self-healing, quite a high incidence of delayed and nonunion of long bone fractures is recorded, also due to concomitant factors such as diabetes, osteoporosis, metabolic diseases, infection, and smoking [4]. Actually, the healing process was shown to slow down with advancing age, possibly due to a lower number of mesenchymal stem cells, or a decrease in their mitogenic potential, or to a poor response to therapeutic interventions [5].

Conventional treatments to heal nonunions include autologous bone graft, combined with internal/external fixation, synthetic bone substitutes, including resorbable calcium phosphates, and distraction osteogenesis, too. Adjuvants to these treatments, such as bone morphogenetic proteins, platelet-rich plasma, and low-intensity pulsed ultrasound, have been employed to promote local biology [6, 7].

Cell- and/or growth factor-based strategies in bone tissue engineering and regenerative medicine are increasingly applied as an alternative to standard surgery, with often substantial benefit for patients [8, 9]. The efficacy of MSC to boost local healing has been proved in different clinical settings, and positive results have been reported using a concentrated bone marrow (BM) aspirate [10, 11], even if more benefits may be obtained from expanded mesenchymal cells [12]. Both strategies aim at enhancing the proportion of active mesenchymal cells, since it is known that the skeletal progenitors in the BM aspirate are rather few (1 in 10^8^ cells), with their number and quality steadily declining with age [13], while their number does matter in the healing process of bone defects [14].

The paracrine activity of MSC, with a release of cytokines, chemokines, and other active molecules, has been recognized as an effector of tissue repair since 20 years and recently described as a major mechanism in bone regeneration [15, 16]. However, the complex network of molecules and factors acting at the healing site prevents an easy identification, and, more importantly, these measurements are invasive and cannot be used in humans [17].

Indeed, the efficacy of the regenerative therapies, that is, bone formation, is currently assessed mostly by X-ray and microcomputed tomography imaging, as well as clinical examination, while other indices, possibly quantitative measurements, are required. The identification of biological markers to help in monitoring the treatment efficacy might facilitate the patient management. By serology, the level of molecules produced at the healing site, to enter circulation afterwards, may be measured and hopefully provide a biological monitoring of the fracture consolidation. However, no studies on this issue are available so far.

In the search for “signals” of MSC regenerating ability, we evaluated prospectively a cohort of patients affected by long bone delayed union or nonunion enrolled in a “Seventh Framework Program” (FP7) European project and treated with a regenerative therapy with expanded autologous MSC plus a synthetic bone substitute. We analyzed changes of a panel of serum bone turnover markers (BTM), commonly used for monitoring the remodelling process [18–20], which were measured at fixed time points according to the schedule of the clinical protocol. We aimed to search for relation, if any, with the treatment outcome, that is, fracture consolidation by clinical and radiological assessment up to one year.

## 2. Patients and Methods

### 2.1. Study Participants and Treatment

In this study, patients affected by delayed union or nonunion of the long bones, aged 18–65 years, were recruited in a prospective, controlled, multicentric, phase I/IIa, and interventional clinical trial entitled “Evaluation of Efficacy and Safety of Autologous MSCs Combined to Biomaterials to Enhance Bone Healing (OrthoCT1)” (ClinicalTrials.gov identifier: NCT01842477).

The inclusion criteria were the following: age 18 to 65, both sexes; delayed union or nonunion at least 3 months from a traumatic, isolated, closed or open, and diaphyseal or metaphysodiaphyseal fracture of long bone. The exclusion criteria were the following: pregnant or breast-feeding women, women who are of childbearing age and not practicing adequate birth control, participation in another therapeutic trial in the previous 3 months; delayed union or nonunion related to iatrogenic causes; segmental bone loss requiring specific therapy (bone transport, large structural allograft, megaprosthesis, etc.); vascular or neural injury; other fractures causing interference with weight-bearing; infection of skin, soft tissue, bone, or any remote infection, that is, dental, pulmonary, and gynecological; visceral injuries of diseases interfering with callus formation (cranio-encephalic trauma, etc.); history of bone harvesting on iliac crest contraindicating bone marrow aspiration; corticoid or immunosuppressive therapy more than one week in three months prior to study inclusion; history of prior or concurrent diagnosis of HIV, hepatitis B, or hepatitis C infection; history of neoplasia or current neoplasia in any organ; insulin-dependent diabetes; obesity (BMI > 30); autoimmune inflammatory disease; and current treatment by bisphosphonate or stopped in the three months prior to inclusion.

**Figure 1:**
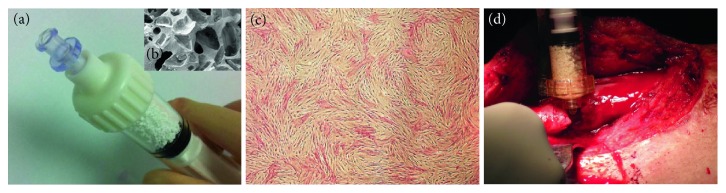
(a) Syringe containing the 1-2 mm MBCP+ granules (5 ml) and (b) scanning electron microscopy picture of the material macroporosity (×30 magnification). (c) Monolayer of bone marrow-derived mesenchymal stromal cells after expansion (alkaline phosphatase cytochemical staining, ×4 magnification). (d) The MSC/MBCP+ mixture is injected into the nonunion fracture site.

The regenerative approach consisted in a minimally invasive administration of autologous bone marrow cells expanded in Good Manufacturing Practice (GMP) facilities (Fondazione IRCCS Ca' Granda Ospedale Maggiore Policlinico, Milano, Italy; Établissement Français du Sang (EFS), Toulouse, France; EFS, Creteil, France; Transfusion Medicine Institute of Ulm, Germany; and Cell Production Unit at Hospital Puerta de Hierro-Majadahonda, Madrid, Spain) and surgically implanted with synthetic biphasic calcium phosphate, that is, 20% hydroxyapatite (HA) and 80% beta tricalcium phosphate (*β*-TCP), in 1-2 mm granules (MBCP+, Biomatlante SA, Vigneux-de-Bretagne, France) (Figures 1(a) and 1(b)).

Bone marrow harvesting was performed in an operating room under anesthesia, from the posterior iliac crest, with a trocar by cutaneous puncture. Bone marrow was aspirated by fractions of 2–4 ml in 20 ml heparin-prefilled syringes, then transferred to the GMP facility, following standard procedures for the transport and cell expansion previously determined [21, 22] (Figure 1(c)).

A standard procedure was applied during the implantation surgery. In the operating room, 200 × 10^6^-expanded MSC were mixed with 10 cc of MBCP+ granules for 60 minutes, then implanted in the bone defect by using two 5 cc syringes (Figure 1(d)). Preclinical studies showed that less than 5% BM-MSC remained in the supernatant or attached to the container after 60 minutes and that cells on the calcium phosphate granules were capable of forming bone [23].

**Figure 2:**
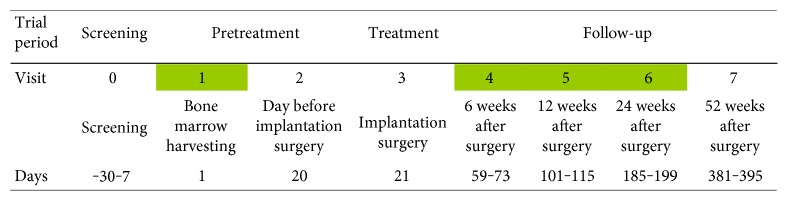
Time course of the OrthoCT1 protocol with the time points of blood collection in green.

The progression of bone healing was evaluated at 6, 12, and 24 weeks after implantation (Figure 2), by clinical and radiological assessment, including visual assessment score (VAS) for pain, digital X-ray (XR), and helicoidal computed tomography (CT) imaging, functional recovery (full weight-bearing gait), and related local complications. Main outcome measure was the time to consolidation, that is, 3 out of 4 or 2 out of 3 cortices on lateral and frontal views, with imaging confirmed bone bridging [24].

### 2.2. Blood Collection and Sample Storage

Each centre participating to the clinical trial collected blood samples as scheduled, that is, at the time of bone marrow harvesting from the iliac crest (visit 1) and, postoperatively, that is at 6 (visit 4), 12 (visit 5), and 24 weeks (visit 6) after surgery for autologous MSC/MBCP+ insertion at the fracture site (Figure 2). A fasting morning blood collection was performed, whenever possible. The blood samples were centrifuged within 2 hours from collection, at 1000 g for 15 minutes at room temperature. The serum was transferred into 12 cryotubes (0.5 ml of serum per cryotube) tagged with a small label showing the project name, acronym of the clinical centre, visit number, date, and patient's inclusion number. The cryotubes were frozen at −20 or −80 degrees Celsius within 4 hours from centrifugation. Later, six frozen cryotubes were sent to the Orthopedic Pathophysiology and Regenerative Medicine Unit of Rizzoli Orthopedic Institute, where all the immunoenzymatic assays were performed.

### 2.3. Immunoenzymatic Assay of BTM

The laboratory personnel performing the immunoenzymatic assay was unaware of the source of the samples, which were marked with numerical codes. The BTM circulating levels were measured using commercially available reagents based on a sandwich enzyme immunoassay technique, following the manufacturer's protocols, with each sample tested in duplicate.  Table 1 shows the list of the BTM selected with a brief description of the source of reagents and the technical notes.

### 2.4. Calculations and Statistical Analysis

Statistical analysis was performed using the StatView 5.0.1 software (SAS Institute Inc., Cary, NC). Data have been expressed as mean ± standard error of the mean of analyte concentration at the different time points and as changes from the baseline value measured at bone marrow harvest. The changes over time have been calculated as follows:

At visit 1 (BM harvesting): 1 − [BTM visit 1/BTM visit 1] = 0 (baseline).
At visit 4: 1 − [BTM visit 4/BTM visit 1].
At visit 5: 1 − [BTM visit 5/BTM visit 1].
At visit 6: 1 − [BTM visit 6/BTM visit 1].

A nonparametric paired analysis of the data (Wilcoxon signed-rank test) was applied patient by patient to detect changes of BTM over time, from visit 1 to visit 6. The Mann–Whitney *U* test was used to highlight significant differences between two independent variables. *P* values < 0.05 were considered as statistically significant.

## 3. Results

### 3.1. Clinical Outcome

Blood samples were collected from twenty-six subjects admitted to the clinical trial, whose demographic and clinical characteristics are shown in Table 2. Two patient dropped out after visit 1 and visit 5.

A satisfactory bone healing was obtained in 23 out of the 24 patients, while one patient without a complete healing was scored as a “failure” at visit 6. Nine subjects reached a good consolidation already at visit 5, and they were considered as the “early consolidation” group.

**Figure 3:**
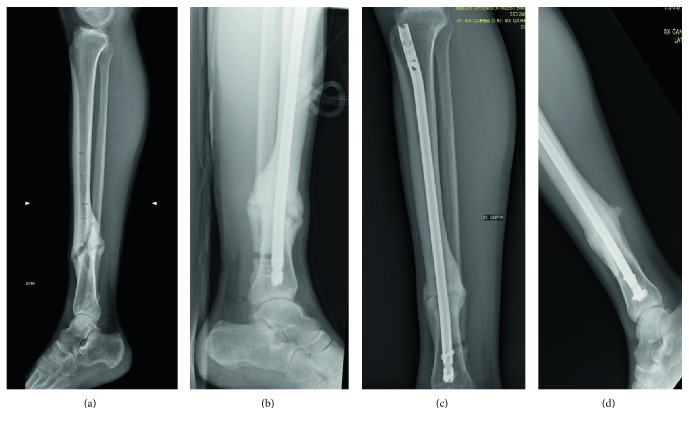
Representative radiological images (lateral view) of a male, 19 years, with irregular complex tibia fracture (1/3 distal diaphysis) not healed after 12 months from trauma; (a) visit 1: pretreatment, day before surgery; (b) visit 3: after implantation surgery; (c) visit 6 at 24 weeks: callus with the same density as cortical; (d) visit 7 at 52 weeks: consolidation.

Representative radiological views are given in Figure 3.

### 3.2. Significant Changes in BTM Levels Are Observed after the Regenerative Treatment

Circulating levels of BTM have been measured in all patients at visit 1, but to highlight the changes over time, we considered only the twenty-four patients with prospective evaluation. At each time point, BTM values were not normally distributed, and a high variability was observed for all markers. Except for PICP and OC, either intact or cleaved, at visit 1, BTM levels tended to be higher than reference values of healthy individuals (Table 1). To verify if significant changes of BTM were induced by implantation of expanded MSC/MBCP+, a patient-by-patient comparison was made (Table 3). After 6 and 12 weeks from surgery, a significant increase in BAP concentration was observed, while CTX and int-OC decreased. Also N-mid OC and PICP decreased after 6 weeks, while a significant change of RANKL was detected after 12 weeks. At 24 weeks, concentrations of all BTM were similar to those observed before treatment.

### 3.3. Some BTM Correlate with Radiological Findings at the Same Time Point

The BTM changes have been calculated for each patient and expressed as previously described, that is, as a ratio between serum concentrations measured at each time point and at baseline (visit 1).

**Figure 4:**
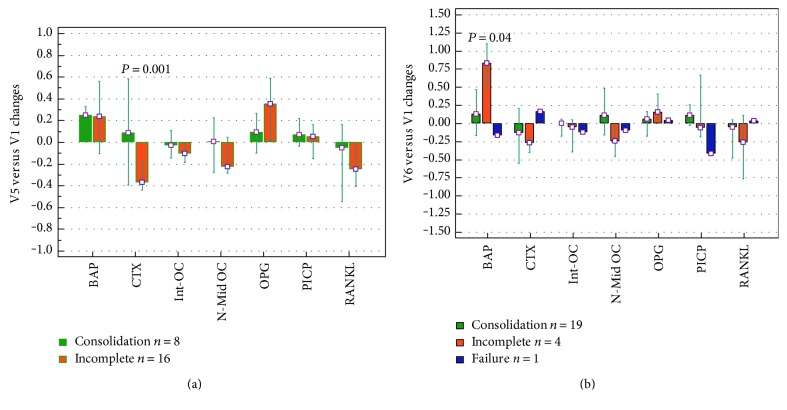
The bars show the median and range (25th to 75th percentile) of BTM changes at visit 5 (a) and visit 6 (b). For each bone marker, the variation at 12 weeks (visit 5) and 24 weeks (visit 6) has been related to the healing status (consolidation, incomplete, and failure) as resulting from imaging at the same time points. BAP: bone alkaline phosphatase; CTX: cross-linked C-telopeptide of type I collagen; int-OC: intact osteocalcin; N-mid OC: N-terminal/midregion osteocalcin; OPG: osteoprotegerin; PICP: C-terminal propeptide of type I procollagen; RANKL: receptor activator of nuclear factor kB ligand; V1: baseline; V5: visit 5; V6: visit 6.

A relationship between BTM changes at 12 and 24 weeks and the healing status by imaging at the same time points was searched. CTX variation was over the baseline level in patients who exhibited a bone bridging at 12 weeks (Figure 4(a)), while decreased in subjects with incomplete consolidation. At 24 weeks, the BAP increase found in the most part of patients was less pronounced in cases with a complete healing (Figure 4(b)).

### 3.4. BTM in Patients with “Early Consolidation” Change with a Different Time Course

**Figure 5:**
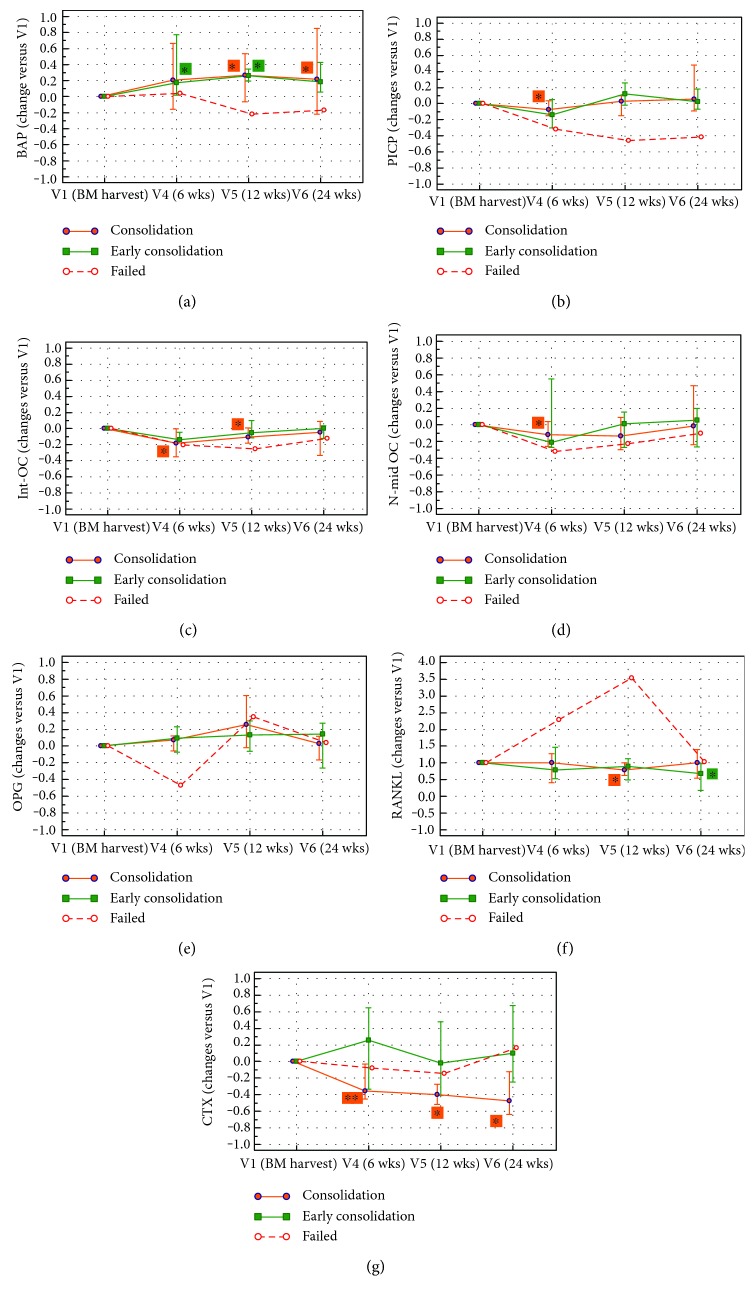
The graphs show the median and range (25th to 75th percentile) of BTM changes from visit 1 (V1), chosen as the baseline value, to visit 6 (V6). For each bone marker, the variation index recorded during the follow-up has been considered according to the final outcome. (a) BAP, bone alkaline phosphatase; (b) PICP, C-terminal propeptide of type I procollagen; (c) int-OC, intact osteocalcin; (d) N-mid OC, N-terminal/midregion osteocalcin; (e) OPG, osteoprotegerin; (f) RANKL, receptor activator of nuclear factor kB ligand; (g) CTX, cross-linked C-telopeptide of type I collagen.

The relationship between BTM changes and primary outcome, that is, fracture consolidation by clinical and radiological assessment up to one year, is shown in Figure 5. At this final time point, 23 out of 24 patients showed a positive outcome, with 8 patients scored as “early consolidation” and 15 patients with positive outcome within one year.

In patients with an “early consolidation,” the BAP level was significantly increased already at 6 weeks, to further increase at 12 weeks, while in patients who consolidated within one year, a statistically significant BAP increase was observed afterwards, that is, at 12 and 24 weeks.

PICP, int-OC, and N-mid OC were significantly decreased at 6 weeks in patients healed after 24 weeks, to increase afterward, with changes not significantly different from baseline values. OPG was increasing over time, but no significant changes were detected, while RANKL was reduced at 12 weeks in patients who consolidated within one year and at 24 weeks in patients with “early consolidation.” However, no significant differences were found between the group of 8 patients who healed within 12 weeks (early consolidation) and the 15 subjects who healed later. Figure 5 also shows the variations versus baseline observed in the single patient who experienced a treatment failure. This patient had a remarkable decrease of BAP and PICP at all time points, while OPG dropped after 6 weeks and RANKL increased dramatically up to 12 weeks.

## 4. Discussion

As recently reported, 5–15% of bone fractures end in impaired bone healing, with additional surgery requirements, even if the nonunion incidence varies significantly depending on the anatomic site and the clinical criteria used to define nonunion. High-energy trauma, inappropriate fracture fixation, large bone loss, low blood supply, and infection have been reported as main reasons for nonunion [25, 26]. The rather high incidence of fracture-delayed unions or nonunions has opened the way to “orthobiologics,” including mesenchymal stromal cells, as well as growth factors or anabolic drugs to accelerate consolidation and prevent or treat delayed unions or nonunions [27, 28].

Following any MSC-based therapeutic intervention on bone, the amount of tissue that is regenerated cannot be reliably predicted. As remarked by Ginis et al., radiological images may overestimate the extent of bone formation during regeneration, but the identification of “true” new bone at the site of regeneration, as obtained by histology and histomorphometry in “in vivo” models, is not feasible in humans [29]. Biomarkers of the activity of bone remodeling may provide additional information beyond radiographic assessments, but to date, a biological factor useful to the clinical monitoring of bone healing has not been identified [30]. Bone turnover markers are routinely used in the diagnosis or treatment monitoring of several bone diseases, such as osteoporosis or Paget's disease, and shown to correlate with clinical findings and bone imaging [31]. In addition, they have been studied also for monitoring the healing process of fractures and for the early detection of fracture-healing disturbance [32–34].

In this study, we hypothesized that circulating BTM could reflect the healing process induced by the regenerative boost of expanded MSC combined with calcium phosphate and that changes of BTM over time could predict the treatment outcome, that is, the consolidation of the bone lesion.

In order to accomplish our goal, a panel of seven markers belonging to the three main categories of BTM, that is, markers of bone formation (BAP, PICP, intact-OC, and N-mid OC), bone resorption markers (CTX, intact-OC, and N-mid OC), and osteoclast regulatory proteins (OPG and RANKL), was selected [18–20, 32–35].

BAP is expressed early during the differentiation of mesenchymal progenitors into osteoblasts, and its circulating levels seem to be directly related to the number and differentiation state of osteoblasts [32]. PICP directly reflects the formation rate of type I collagen, and often, after an initial decrease in PICP levels, the turnover of type I collagen in fractured patients shows a gradual rise over time [36]. To complete the picture of the matrix synthesis, OC, a noncollagenous protein released by differentiated osteoblasts, was measured. OC is incorporated into the organic bone matrix, and only a small fraction is circulating. Even though it is widely accepted as a marker of osteoblastic activity [37], during bone resorption, the OC entrapped in the mineralized matrix is released so that serum concentration also reflects the osteolytic process; hence, OC is considered a marker of bone turnover, rather than a specific marker of bone formation. Intact OC (amino acids 1–49) is unstable due to protease promoting the cleavage between amino acids 43 and 44, while the N-mid fragment (amino acids 1–43) is considerably more stable [38].

OPG and RANKL are regarded as main regulators of osteoclast differentiation and function. The pro-osteoclastogenic factor RANKL is released by mature osteoblasts in order to activate the resorption and to initiate the remodeling cycle in basic multicellular units. Bone-forming cells produce also OPG, which acts as a decoy receptor and modulates RANKL activity, in order to maintain a balance between bone formation and bone resorption [39].

During the callus remodeling, an increase of markers of bone resorption is expected [33]. As a marker, we selected CTX that derives from the digestion of type 1 collagen mediated by cathepsin K produced by osteoclasts [32, 40].

All the markers, but CTX, are directly or indirectly related to the osteoblast differentiation, and therefore, they could be useful to follow in vivo the bone regeneration promoted by cell therapies based on mesenchymal stromal cells.

According to the final result of the clinical trial, only one of the recruited patients did not reach a stable consolidation within one year from the regenerative treatment. As a consequence, no chance to calculate the predictive value of BTM was given. However, our data allowed to verify if BTM levels (i) changed after the regenerative therapy based on ex vivo expanded MSC, (ii) correlated to the imaging results at visit 5 and visit 6, and (iii) differed in patients who had an “early consolidation.”

Actually, BTM changed over time, and all variations, but OPG, were statistically significant at one or more time points, with a peak at 6 weeks from surgery when 5 out of 7 markers showed significant changes. An increase in BAP concentration was observed, while PICP, CTX, and both OC tended to decrease. This picture was maintained at the midpoint (12 weeks) with the addition of a significant decrease in RANKL levels, but at 24 weeks, all BTM returned to values observed at baseline.

We expected that our results could reflect the kinetics of BTM during the fracture healing, even if literature data on this topic are quite controversial. The BAP increase, from 4 weeks after fracture up to one year, is confirmed by a number of studies [33, 41–44]. However, other BTM such as CTX, PICP, and OC, have been shown to increase following an initial fall after fracture [32, 33], but we did not find this result in our case series. Few studies have analyzed the serum levels of RANKL and OPG during the fracture healing process, and a high variability of results was found [45–47]. Therefore, our findings suggest that bone repair steps following the physiological fracture healing or a regenerative treatment differ to some extent.

Then, it was verified whether there was a relationship between BTM changes at 12 and 24 weeks and the healing status evaluated by XR or CT at the same time points, and we found that CTX and BAP correlated with the imaging results. CTX variation was over the baseline value in patients who were already healed at 12 weeks, while a negative value was recorded in patients with an incomplete consolidation. At 24 weeks, a BAP increase was found in the most part of patients, but the rise was less pronounced when the consolidation was reached. These findings reflect what is expected at the end of the bone healing process, when the osteogenic phase is progressively silenced and the remodeling phase prevails [34, 48].

Finally, we try to understand if the overall BTM variation along the healing course was different in patients who had an early consolidation. A similar overall BTM trend was shared by patients healed at 12 weeks and those with later consolidation, but significant changes were displayed already at 6 weeks by the “early” ones. Based on these findings, we can suggest that the BTM kinetics is influenced by the healing pace, but unfortunately, our hypothesis is not supported by other studies dealing with a relation between BTM levels and the timing of healing. However, it cannot be excluded that the rate of callus formation and healing may be one of the reasons of the high individual variability of BTM observed in response to fracture, as remarked in a number of papers [25, 32, 49].

In the single patient who did not reach the consolidation at the end point, the variations of BAP and PICP, that is, the bone formation markers, differed consistently from the other subjects. A similar picture is described by authors who reviewed the role and potential of biochemical serum markers as indicators of fracture healing disturbances [33, 34, 36, 50, 51]. In theory, the above molecules could reflect a poor response to the regenerative treatment, but this cannot be ascertained in our study because of the lack of failed cases.

Summarizing, the feasibility and efficacy of the regenerative treatment using a high amount of expanded MSC with an osteoconductive material was the primary objective of this phase I/IIa clinical trial, but some limitations emerge when focusing on the role of BTM changes as “signals” of the MSC regenerating ability. Taken together, our results prove that BTM monitoring is practicable and suggests a possible correlation with clinical results, but the real contribution of the transplanted MSC/MBCP+ to the observed BTM changes can be only hypothesized. In fact, BTM could vary also in the absence of MSC implantation, but unfortunately, a control group treated with a standard therapy without biological supplementation was not included in the clinical protocol.

In addition, while the regenerative treatment was homogeneous for all patients, that is, same MSC dose, biomaterial volume, and MSC/biomaterial ratio, the clinical heterogeneity, that is, the type of fracture, mobility, and fixation devices, could influence the BTM levels. Anyway, when considering all variables, the number of patients recruited in our pilot study was not large enough to obtain a statistical evidence.

## 5. Conclusion

Nonunions are among the leading indications for cell therapy bone repair in everyday practice [30]. A nonsubjective assessment of bone healing, possibly at an early stage, is eagerly awaited by clinicians to predict treatment outcome, but currently, there is no reliable way to predict which patients will benefit from regenerative approaches. We evaluated a panel of BTM that could reflect the healing rate, to be used as a supplemental marker for monitoring or predicting consolidation. Our data show that BTM (i) change after the MSC/MBCP+ regenerative therapy, (ii) correlate to the imaging results at certain follow-up time points, and (iii) differ in patients who had an “early consolidation.” Yet, despite the remarkable differences in bone formation markers, the predictive value of BTM changes cannot be determined, since a single failure was observed, that is, about 5% of the treated cases. A recently approved trial with a large case series and a control population treated with standard techniques will allow to overcome the limits of the study and to assess if BTM are useful for monitoring cell therapy efficacy in bone nonunions.

## Figures and Tables

**Table 1:** Source of the immunoenzymatic assay kits, sensitivity, precision, and reference values from healthy individuals.

Marker	Commercial name (source)	Detection limit	Intraassay CV Min–max (%)	Interassay CV Min–max (%)	Female (postmenopausal) Mean ± SEM Median Min–max	Female (premenopausal) Mean ± SEM Median Min–max	Male Mean ± SEM Median Min–max
BAP (*μ*g/l)	Ostase BAP (Immunodiagnostic systems)	0.7	2.6–6.5	3.7–6.1	14.53 ± 4.21	9.0 ± 2.98	13.13 ± 3.8
					13.2	8.78	12.3
					8.0–22.4	4.0–14.30	7.0–20.1

CTX (*μ*g/l)	Serum crosslaps (Immunodiagnostic systems)	0.02	1.8–3.0	2.5–10.9	0.64 ± 0.36	0.38 ± 0.19	0.39 ± 0.19
					0.44	0.29	0.29
					0.12–1.35	0.11–0.74	0.12–0.75

int-OC (*μ*g/l)	hOST EASIA (DIAsource ImmunoAssays)	0.08	3.1–4.7	3.5–5.6	15.00 ± 5.77		
					15.00		
					5.0–25.0		

N-mid OC (*μ*g/l)	N-mid Osteocalcin (Immunodiagnostic systems)	0.5	1.3–2.2	4.2–5.1	31.43 ± 12.43	19.90 ± 7.47	23.40 ± 9.19
					26.5	17.40	19.8
					12.8–55	8.40–33.9	9.6–40.8

OPG (pmol/l)	Osteoprotegerin (Biomedica)	0.07	2-3	3–5	—		
					2.7		
					—		

PICP (*μ*g/l)	MicroVue CICP EIA (Quidel)	0.2	5.0–7.2	Not declared	116.00 ± 27.14		
					116		
					69.0–163		

RANKL (pg/l)	Free soluble RANKL (high sensitivity) (Biomedica)	0.01	≤5%	≤3%	—		
					0.14		
					—		

BAP: bone alkaline phosphatase; CTX: cross-linked C-telopeptide of type I collagen; int-OC: intact osteocalcin; N-mid OC: N-terminal/midregion osteocalcin; OPG: osteoprotegerin; PICP: C-terminal propeptide of type I procollagen; RANKL: receptor activator of nuclear factor kB ligand.

**Table 2:** Demographic and clinical characteristics of patient cohort.

Number of subjects evaluated at Rizzoli Orthopedic Institute	*N* = 26
Age	
Mean ± standard deviation	39.6 ± 14
Median	41.5
Range	19–65
Gender	
Males	*N* = 15
Females	*N* = 11
Site of lesion	
Femur	*N* = 11
Tibia	*N* = 9
Tibia & Fibula	*N* = 2
Humerus	*N* = 3
Not recorded	*N* = 1
Surgery and Osteosynthesis^a^	*N* = 25
Nail	*N* = 15
Plate	*N* = 9
External fixator	*N* = 1
Patients with complete blood sample collection (4 time points)	*N* = 23
Patients prospectively evaluated (at least 2 time points)	*N* = 24
Final outcome	
Consolidation	*N* = 23
Failure	*N* = 1

^a^One patient was recruited but not operated on.

**Table 3:** Circulating BTM levels (concentration/litre) measured in all patients.

	V1 (BM harvest) *N* = 26	V4 (6 wks) *N* = 24	V4 versus V1	V5 (12 wks) *N* = 24	V5 versus V1	V6 (24 wks) *N* = 24	V6 versus V1
	Mean ± SEM Median Min–max	Mean ± SEM Median Min–max	*P* value	Mean ± SEM Median Min–max	*P* value	Mean ± SEM Median Min–max	*P* value
BAP (*μ*g/l)	20.11 ± 2.63	23.71 ± 3.24	**0.035**	23.55 ± 3.22	**0.011**	21.10 ± 1.99	0.412
	14.61	20.81		19.73		20.52	
	7.71–66.74	9.82–88.16		9.53–86.48		8.01–51.80	

CTX (*μ*g/l)	0.81 ± 0.10	0.62 ± 0.08	**0.028**	0.65 ± 0.10	**0.014**	0.62 ± 0.08	0.073
	0.75	0.51		0.50		0.56	
	0.14–2.50	0.28–2.15		0.20–2.11		0.13–1.84	

int-OC (*μ*g/l)	8.78 ± 0.56	7.28 ± 0.48	**0.001**	8.06 ± 0.62	**0.036**	8.58 ± 0.75	0.421
	9.26	7.85		8.18		8.47	
	3.82–12.96	3.51–10.91		3.80–13.86		3.22–14.24	

N-mid OC (*μ*g/l)	14.88 ± 1.91	13.13 ± 1.86	**0.016**	13.37 ± 2.12	0.290	14.42 ± 1.89	0.330
	13.76	10.62		11.38		11.81	
	1.35–47.08	4.51–42.94		1.91–51.30		2.28–34.67	

OPG (pmol/l)	5.96 ± 0.75	5.81 ± 0.79	0.241	6.55 ± 0.87	0.056	5.48 ± 0.70	0.465
	4.51	5.12		5.44		4.37	
	1.63–16.11	1.53–19.48		1.45–18.90		1.62–14.55	

PICP (*μ*g/l)	121.26 ± 16.55	106.47 ± 15.33	**0.016**	113.44 ± 12.88	0.987	142.81 ± 18.48	0.570
	97.23	88.14		105.83		106.99	
	51.46–466.34	36.40–418.68		40.79–357.58		65.02–385.51	

RANKL (pg/l)	0.19 ± 0.03	0.21 ± 0.06	0.455	0.14 ± 0.02	**0.022**	0.15 ± 0.03	0.149
	0.20	0.14		0.14		0.08	
	0.00–0.46	0.00–1.33		0.00–0.45		0.00–0.62	

V1: visit 1; V4: visit 4; V5: visit 5; V6: visit 6; BAP: bone alkaline phosphatase; CTX: cross-linked C-telopeptide of type I collagen; int-OC: intact osteocalcin; N-mid OC: N-terminal/midregion osteocalcin; OPG: osteoprotegerin; PICP: C-terminal propeptide of type I procollagen; RANKL: receptor activator of nuclear factor kB ligand.
